# Acute uveitis caused by abnormal glucose and lipid metabolism: a case report

**DOI:** 10.1186/s12886-023-02997-z

**Published:** 2023-06-12

**Authors:** Zhaojing Bi, Yan Liang, Shujun Liu, Yuanbin Li

**Affiliations:** 1 School of Clinical Medicine, Weifang Medical University, Shandong, China; 2 Department of Ophthalmology, The Affiliated Yantai Yuhuangding Hospital of Qingdao University, Shandong, China

**Keywords:** Acute uveitis, Hyperlipidemia, Diabetes mellitus, Blood-aqueous humor barrier, Anterior chamber reaction

## Abstract

**Purpose:**

This report describes a rare case of acute uveitis with severe anterior chamber inflammation due to abnormal glucose and lipid metabolism.

**Case presentation:**

A 31-year-old male patient complained of redness in the right eye with decreased visual acuity for 3 days. Ocular examination revealed a milky white clouding of the right anterior chamber of the eye. Two clusters of yellowish-white exudates were visible on the surface of the iris in the upper nasal and temporal areas in addition to elevated intraocular pressure. He had a previous diagnosis of type 2 diabetes mellitus (T2DM). Laboratory tests suggested hyperlipidemia and ketoacidosis. After admission, topical glucocorticoids, mydriasis, and intraocular pressure-lowering drugs combined with hypoglycemic and lipid-lowering therapy and fluid replacement therapy were given immediately. After 10 days of treatment, the uveitis and systemic condition of the right eye were effectively controlled and improved.

**Conclusion:**

Abnormal glucose and lipid metabolism leads to impairment of the blood-aqueous barrier, which causes a severe uveitis response in the anterior chamber. After the use of topical steroids and mydriatic eye drops combined with systemic hypoglycemic and lipid-lowering interventions, the condition was significantly relieved.

## Background

Uveitis is an inflammation of the uvea, including the iris, ciliary body, and choroid, with frequent involvement of the retina and vitreous [1]. There are also many cases of uveitis of unknown etiology. It causes 5–10% of all visual impairments worldwide [2]. Studies have found that [3] acute uveitis is more common in diabetic patients with poor glycemic control and may be further exacerbated by damage to the blood-aqueous humor barrier in patients with comorbid hyperlipidemia [4]. When the integrity of the blood-aqueous barrier is compromised and there is coexisting hyperlipidemia, the likelihood of lipid leakage into the aqueous humor may be increased [4, 5]. This is similar to the case reported here; however, it is rare for such a severe anterior chamber reaction to occur due to abnormal glucose and lipid metabolism.

## Case presentation

**Fig. 1:**
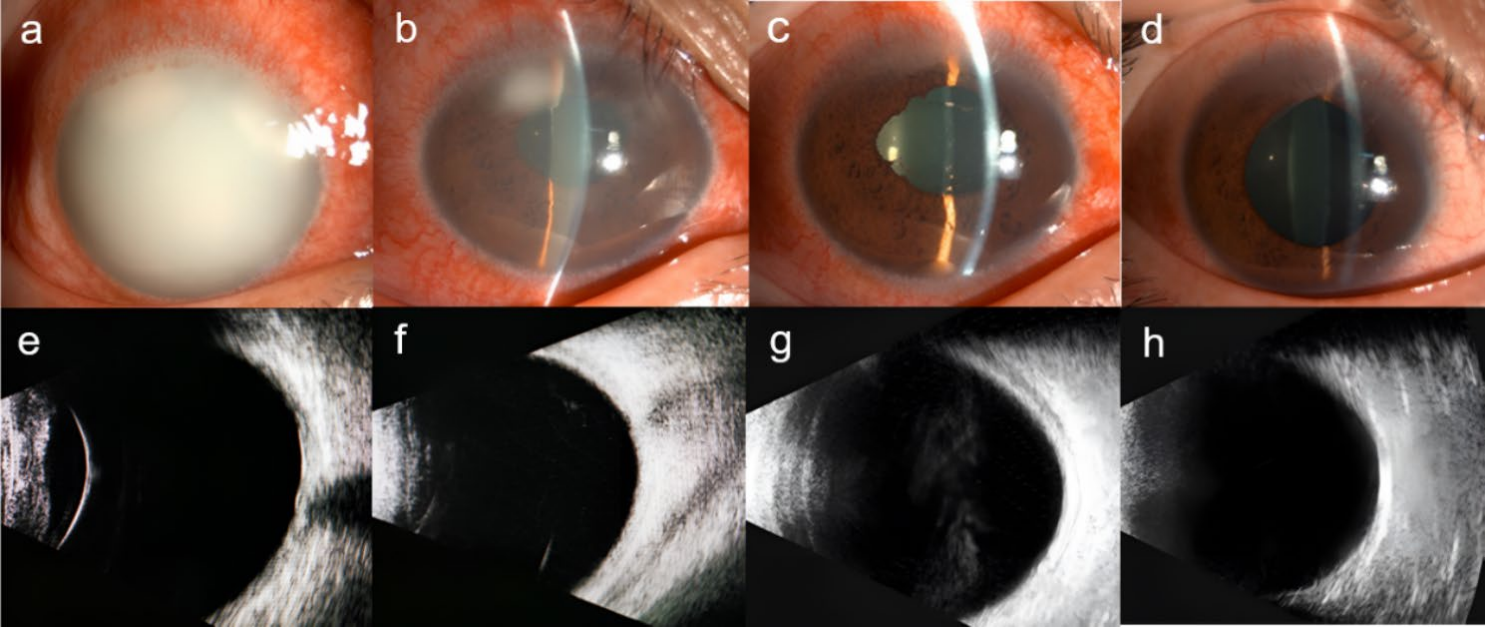
OD changes in uveitis. The top row shows eye segment photographs, and the bottom row shows eye ultrasound images on (**a**)(**e**). day 1, (**b**)(**f**). day 2, (**c**)(**g**). day 3, and (**d**)(**h**). day 10 of admission (discharge from hospital)

**Fig. 2:**
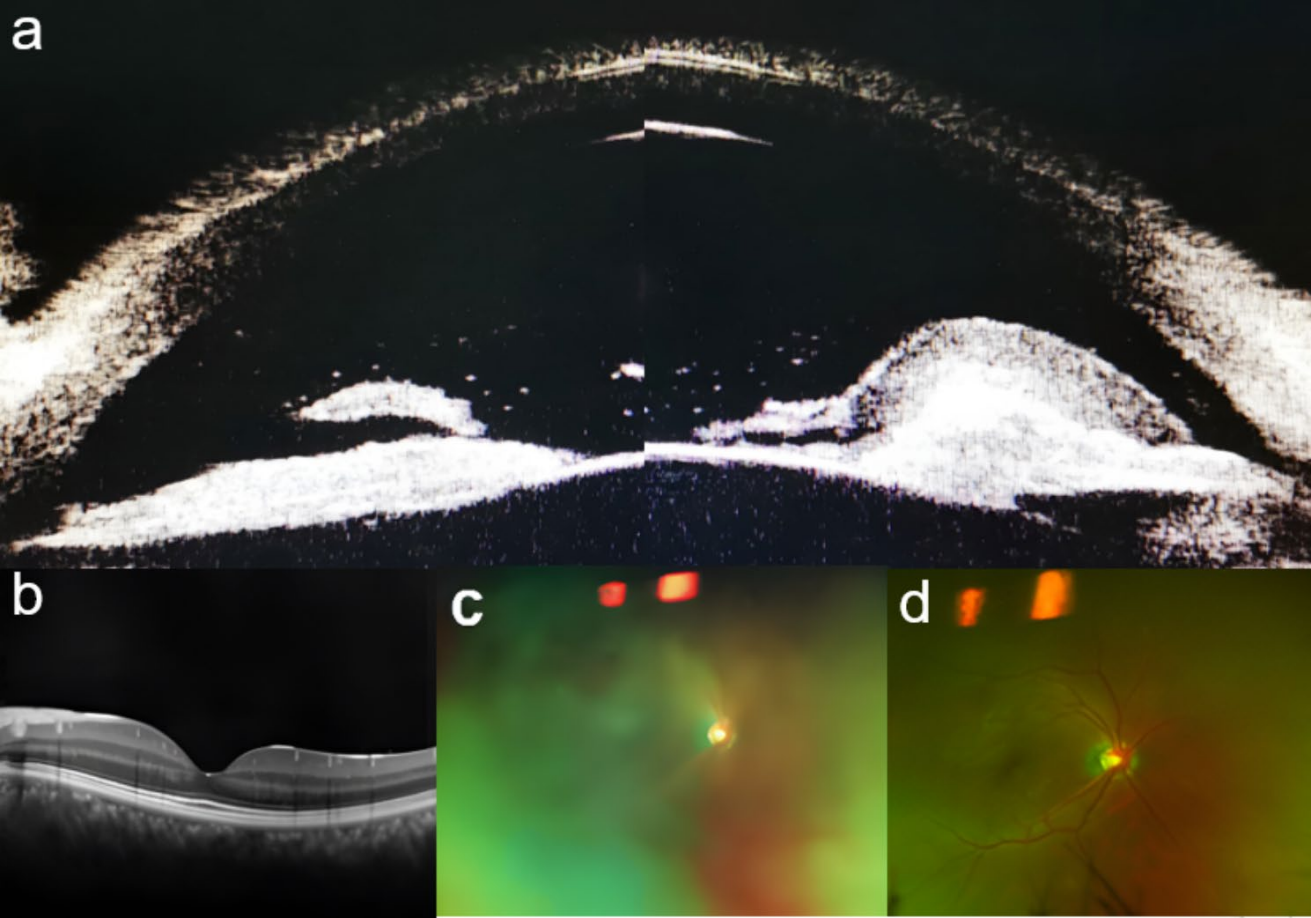
OD-related ancillary tests (**a**). Day 1 UBM: severe anterior chamber clouding and a coalescence of exudate adhering to the iris surface (yellow arrow) can be seen. (**b**). Day 3 OCT: no significant abnormalities are shown. (**c**). Day 3 fundus photograph: vitreous clouding is observed with no obvious abnormalities in the fundus. (**d**). Day 10 fundus photograph: No significant abnormalities are shown

The patient, a 31-year-old male, was admitted to our ophthalmology clinic with “redness in the right eye with loss of vision for 3 days” due to “acute anterior uveitis in the right eye”. His past medical history included type 2 diabetes mellitus (T2DM) for 3 years without systematic medication treatment but no history of hereditary eye disease or ocular trauma. Ophthalmologic examination after admission included visual acuity in the right eye (Hand Motion/Before Eye) and intraocular pressure (27 mmHg) and revealed mixed conjunctival congestion in the right eye, a cloudy opalescent appearance of the cornea, an intact epithelium, no ulcerative foci, cloudy a upper nasal and upper temporal iris surface, and two yellowish-white exudate adhesions; the remaining intraocular structures could not be seen (Fig. 1a). Ultrasound biomicroscopy of the right eye showed severe anterior chamber clouding and a coalescence of exudate adhering to the iris surface (Fig. 2a). Ocular ultrasound showed no significant abnormalities (Fig. 1e). Laboratory tests included the fasting glucose level was 14.50 mmol/L (normal: 3.80–6.20 mmol/L), postprandial glucose level between 16 and 22 mmol/L, glycated hemoglobin was 12.6% (4.2–6.2%), bicarbonate was 18.3 mmol/L (21.0–30.0 mmol/L), total cholesterol was 9.41 mmol/L (3.12–5.72 mmol/L), triglyceride was 13.55 mmol/L (0.40–1.70 mmol/L), and erythrocyte sedimentation rate was 65 mm/h (< 20 mm/h). White blood cell count, C-reactive protein, rheumatoid factor, SLE autoantibodies, viral antibodies and HLA-B27-related autoantibodies were within normal levels. Conjunctival sac detection revealed no bacterial or fungal growth. Abdominal ultrasound showed no obvious abnormalities. The patient’s preliminary diagnosis was acute uveitis OD, secondary glaucoma OD, type II diabetes mellitus, and hyperlipidemia.

After admission, the patient was immediately administered 0.1% tobramycin dexamethasone drops Q2H in the right eye, compound tropicamide drops QID in the right eye, 1% brinzolamide drops TID in the right eye, and a 3 mg dexamethasone periocular injection was administered in the right eye in combination with systemic hypoglycemic and hypolipidemic drugs and rehydration therapy. On the second day of admission, the patient’s condition improved significantly, with a visual acuity of 0.2 in the right eye, an IOP of 24 mmHg, corneal transparency, significantly reduced opalescent clouding of the anterior chamber, only small patches of white exudate adhering to the iris surface, a visible iris texture, and mild pharmacological dilatation of the pupil (Fig. 1b and f); therefore, the medication was changed to 1% atropine drops in the right eye BID. The anterior chamber had an aqueous flare (++++), some iris adhesions, a plum-shaped pupil with a diameter of approximately 5 mm, visible slight pigmentation in the anterior lens capsule, and increased vitreous clouding (Figs. 1c and 2c). Ultrasound showed dense punctate hypoechogenicity in the vitreous cavity of the right eye (Fig. 1g) and no significant abnormality in the macula (Fig. 2b); we considered whether the uveitis had involved the vitreous. While controlling stable blood glucose levels, the patient received 20 mg of oral prednisone to control the progression of ocular inflammation.

On the 10th day after admission, the visual acuity of the right eye was 0.6, the cornea was clear, the anterior chamber had an aqueous flare (+), the iris texture was smooth, the pupil was medically dilated, the lens was clear, the vitreous inflammatory clouding was significantly reduced (Fig. 1d and h), the fundus was visible since the optic disc boundary was clear and distinguishable, and the retina was attached (Fig. 2d). The patient was discharged with a stable ocular and general condition. The patient underwent a follow-up examination 1 month later. The best corrected visual acuity (BCVA) was 0.8 in the right eye and 1.0 in the left eye. Both eyes had clear corneas, clear aqueous humor, and stable ocular conditions. Laboratory tests showed that the patient’s blood glucose and blood lipids were normal.

## Discussion

Numerous studies [6, 7] have found that elevated blood glucose levels in diabetic patients cause the upregulation of inflammatory factors, leading to iris capillary endothelial cell edema, reduced vascular resistance and increased vulnerability, while hyperlipidemia caused by disorders of lipid metabolism due to DM affects lipoprotein composition and excessive elevation of triglycerides, leading to prolonged residence time in the endothelium and increased susceptibility to endothelial cell injury, both of which play a role in the destruction of the blood-aqueous humor barrier [8]. Related literature reports that metabolic disturbances occurring in the diabetic state alter the fine structure and function of the endothelial cell plasma membrane, and these changes lead to an increase in endothelial cell permeability in the intraocular capillaries [9]. These changes may increase the chance of inflammatory substance and lipid leakage into the aqueous humor.

In this report, we analyze a rare case of acute anterior uveitis in which the characteristic ocular symptom was the sudden appearance of a large amount of exudate in the anterior chamber accompanied by a sudden decrease in visual acuity. Endophthalmitis is one of the most devastating eye infections and may lead to irreversible blindness in the infected eye within hours or days of symptom onset [10]. According to the patient’s history, auxiliary examinations and changes in signs during treatment, their disease was not consistent with the development and characteristics of endophthalmitis. The patient also had hyperlipidemia in addition to a significant elevation of glycated hemoglobin, and no other causative factors were found that could trigger uveitis. However, we found that the patient showed significant improvement in visual acuity and general condition after treatment for conventional uveitis as well as glucose and lipid control.

Scheen [11] and Gilbert et al. [12] found that lowering elevated glucose and blood lipid levels in some patients may be more conducive to the regression of inflammation. Thus, in our patient, good control of blood glucose and lipids may have helped to reduce ocular inflammation caused by uveitis and may have been an important factor in preventing inflammation recurrence. Our diagnosis of uveitis due to abnormal glucose and lipid metabolism in this case remains hypothetical. We believe that there is a correlation in specific populations of patients with uveitis. At the same time, our study provides a new basis for the rapid clinical diagnosis and treatment of special causes of uveitis.

## Data Availability

All the data supporting the conclusions of this article are included in the present article.

## Abbreviations

T2DM: Type 2 diabetes mellitus
